# Absence of mutations associated with resistance to benzimidazole in the beta-tubulin gene of *Ascaris suum*

**DOI:** 10.1590/0037-8682-0155-2019

**Published:** 2020-03-16

**Authors:** Adalid Palma, Gabriela Matamoros, Denis Escobar, Ana Lourdes Sánchez, Gustavo Fontecha

**Affiliations:** 1 Microbiology Research Institute, Universidad Nacional Autónoma de Honduras (UNAH), Tegucigalpa, Honduras.; 2 Department of Health Sciences, Brock University, St. Catharines, Ontario. Canada.

**Keywords:** Ascaris suum, Benzimidazole, Beta-tubulin gene

## Abstract

**INTRODUCTION:**

Benzimidazoles are commonly used for the control of veterinary nematodes. Resistance to benzimidazoles has been associated with three single nucleotide polymorphisms in the β-tubulin gene of common nematodes. However, these mutations are infrequent in the genus *Ascaris* spp.

**METHODS:**

In order to determine mutations associated with benzimidazole resistance in *Ascaris suum,* worms were collected from slaughtered pigs and a partial region of the β-tubulin gene was sequenced.

**RESULTS:**

All parasites showed the wildtype genotype for codons 167, 198, and 200 of the β-tubulin gene.

**CONCLUSIONS:**

This is the first report of genetic sequences associated with benzimidazole resistance in *A. suum*.

*Ascaris suum* infections are highly prevalent among domestic pigs in all production systems worldwide. In contrast to other helminthiases of veterinary importance, porcine ascariasis has not historically been a clinical priority for veterinarians and pig farmers because the infection seldom causes significant animal health problems. However, the internal damage caused by the transit of the larvae through the liver and lungs on their way to the animal’s small intestine has been associated with decreases in production profitability^1^. 

To reduce the negative consequences of helminthiases on production, farmers use anthelmintic drugs as a routine prophylaxis to control the infections in herds of domestic pigs. However, the long-term use of anthelminthic drugs and mass drug administration programs has led to the selection of drug-resistant veterinary and human parasites^2^. 

Benzimidazoles (BZ) are anthelminthic drugs commonly used for the control of nematodes of veterinary importance. Worldwide, they are the primary treatment of choice because of their safety, low cost, and efficiency. Drugs of the BZ family exert their effect by binding to β-tubulin and blocking the polymerization of microtubules in nematodes cells^3^. Up to three single nucleotide mutations in the β-tubulin gene have been associated with resistance to BZ in superfamilies of either parasitic or free-living nematodes, such as Trichostrongyloidea^4^ ^,^ ^5^, Strongyloidea^6^, Rhabditoidea^7^, Strongyloidoidea^8^, and less frequently in Filarioidea and the Family Trichuridae^9^ ^,^ ^10^. However, these mutations have not been reported in or are not directly associated with BZ resistance in two roundworm superfamilies of the suborder Spirurina: Heterakoidea^11^ ^,^ ^12^, and Ascaridoidea^2^ ^,^ ^9^ ^,^ ^11^ to which the genera *Ascaridia* and *Ascaris* belong, respectively.

Further studies are required not only to better understand the differences among taxa, but to discern the effect of the mutated alleles of the β-tubulin genes in *Ascaris* spp on BZ resistance. In this study, we analyzed a partial sequence of the β-tubulin gene of adult *Ascaris* worms recovered from domestic pigs in order to detect three mutations that have been commonly associated with BZ resistance in other nematodes.

Fifty adult pig-derived *Ascaris* worms were manually collected from the small intestine of 17 pigs slaughtered for commercial purposes in a local abattoir in Tegucigalpa, Honduras in 2017. One to six worms were collected from each animal. Worms were thoroughly washed with a saline solution and stored at room temperature in 70% (v/v) ethanol until further use. DNA was purified using a previously described method^13^. Two separate PCR reactions were carried out to analyze three codons of the β-tubulin gene. The first PCR reaction amplified the gene region containing codon 167, while a semi-nested PCR approach was used to amplify the sequence containing codons 198 and 200^10^ ^,^ ^14^ ^,^ ^15^. All reactions were carried out in a volume of 50 μl, containing 25 μl of 2X Master Mix (Promega Corp.^®^ Madison, WI, USA), 1.0 μl of each primer (10 μM) (Table 1), 2.0 μl of 10 mg/ml acetylated bovine serum albumin and 2.0 μl of DNA (20 ng/μl). For the semi-nested PCR, 1 μl of the first amplification product was used as template. PCR reactions were performed in a Veriti Thermal Cycler (Thermo Fisher Scientific^®^, Waltham, MA, USA) under the conditions described in Table 1. All PCR reactions included an initial denaturation step of 95ºC for 5 minutes and a final extension step of 72ºC for 5 minutes.

**TABLE 1:** Primer sequences and PCR conditions for the amplification of two regions of the β-tubulin gene of *Ascaris* spp.

Codon	Primer	Primer sequence 5’- 3’	Nº of cycles	PCR conditions	Product size (bp)
167	Fwd-Al167	CCGTGAAGAATACCCCGAC	50	94ºC / 45s	388
	Rev-Al167.2	GATGAACGGACAACGTTGC		59ºC / 45s	
				68ºC / 60s	
					
198/200 PCR1	Fwd-Al200/198	AGAGCCACAGTTGGTTTAGATACG	35	95ºC / 60s	
	Rev-Al200/198.1	AGGGTCCTGAAGCAGATGTC		63ºC / 60s	
				72ºC / 90s	
					
198/200 PCR2	Fwd-Al200/198	AGAGCCACAGTTGGTTTAGATACG	35	95ºC / 60s	179
	Rev-Al200/198.2	CAGATGTCGTACAAAGCCTCATT		64ºC / 60s	
				72ºC / 90s	

PCR products were separated on 1.5% agarose gels with ethidium bromide. PCR products were purified and sequenced by Macrogen Inc. (Maryland, USA) using the primers Rev-Al167.2 and Fwd-Al200/198 for codons 167 and 198/200 respectively. Nucleotide sequences were trimmed and manually corrected using the Geneious^®^ 9.1.2 software (Biomatters Ltd. Auckland, New Zealand). The ClustalW tool was used to align sequences. Sequences were deposited in the NCBI database.

Of the 50 adult *Ascaris* worms collected from 17 pigs, six (12%) had previously been classified as putative hybrids (or heterozygotes) between pig- and human-derived *Ascaris* through a PCR-RFLP approach of the nuclear internal transcribed spacer (ITS) ribosomal region^13^. The remaining 46 worms were classified as pig-derived *Ascaris* (*Ascaris suum*). Each hybrid worm was collected from a different animal. All 50 worms (100%) showed the wildtype alleles for the codons 167 (TTC - Phe), 198 (GAA - Glu), and 200 (TTC - Phe) of the β-tubulin gene (Figure 1). 

**FIGURE 1:**
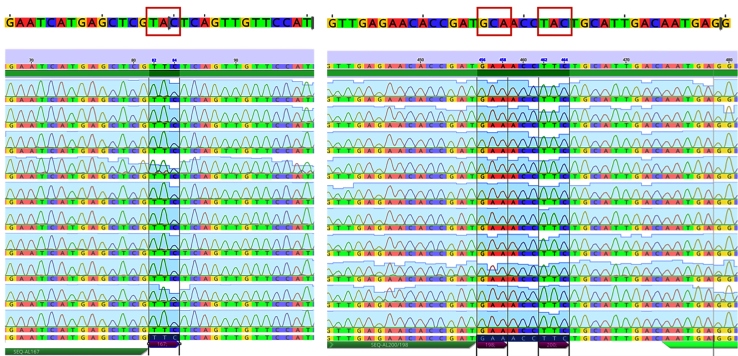
Chromatograms showing codons 167, 198, and 200 from 10 partial β-tubulin sequences of *Ascaris* spp. In the upper portion of the figure, the sequences containing the codons associated with resistance to BZ are highlighted.

BZ resistance in Nematoda has been associated with 3 amino acid substitutions of the β-tubulin gene: a substitution from Phe (TTC) to Tyr (TAC) at codons 167 and 200, and a substitution from Glu (GAA) to Ala at codon 198. Two representative sequences were deposited in the GenBank under the Accession numbers MK296410 and MK296411 for the codons 167 and 198 / 200, respectively. 

To our knowledge, this is the first study analyzing genetic mutations in the β-tubulin gene of *Ascaris* adult worms isolated from domestic pigs. Our results are in agreement with previous studies that were unable to demonstrate the presence of mutations associated with BZ resistance in either *Ascaris lumbricoides* or taxonomically related species. The first study that analyzed codon 200 of the β-tubulin gene of *A. lumbricoides* isolated from three different countries did not find any mutations associated with BZ resistance^9^. A few years later, the same authors reported that codon 167 of *A. lumbricoides* was polymorphic but codons 198 and 200 were monomorphic and 100% wildtype^10^. More recently, a multi-state Brazilian study analyzed more than 600 eggs of *A. lumbricoides* using a PCR-RFLP approach and found no mutations in codons 167 and 198^16^. Similar data were observed when the β-tubulin gene of two species phylogenetically close to *Ascaris*, *Parascaris equorum*, and *Ascaridia galli* (order Spirurina) was analyzed^11^ ^,^ ^12^. These genetic data suggest that species of the order Spirurina may have a limited ability to develop resistance compared to nematodes of the order Strongylida (Superfamily Trichostrongyloidea), such as *Haemonchus*, *Ostertagia*, *Cooperia*, and *Teladorsagia* ^4^ ^,^ ^5^. 

It could be hypothesized that the ability to develop genetic resistance to BZ is directly related to the evolutionary closeness of these genera. Another hypothesis to explain why some species are more or less likely to acquire resistance mutations is based on the number of paralog genes of β-tubulin in each species. Tyden and collaborators described at least two isotypes of β-tubulin in *Parascaris equorum* ^17^ while other authors report that there are at least three closely related β-tubulin genes in *Ascaris suum* ^2^ ^,^ ^4^. This genetic duplication along with the scarce understanding of each paralog’s specific function makes the analysis of this particular gene more complex. In the Trichuridae family, such analysis is facilitated by the fact that only one isotype of the gene has been identified^11^. 

This study has limited information about the geographic origin of the animals and the deworming treatments that they may have received prior to slaughter. This could be causing a bias in the results because if all animals have a similar origin and were subjected to the same treatment and management, they are expected to present the same genetic profile for the β-tubulin gene.

In conclusion, the present study analyzes, for the first time in *A. suum,* three SNPs in the β-tubulin gene associated with BZ resistance. Our findings support the idea that nematodes of the order Spirurina may be less likely to develop genetic resistance to BZ treatment. Nonetheless more research is needed on the genetic variability of the β-tubulin isotypes of these parasites. Future studies including evidence of fecal egg count reduction and *in vivo* efficacy tests are recommended.
